# The Case of Deborah Rice: Who Is the Environmental Protection Agency Protecting?

**DOI:** 10.1371/journal.pbio.0060129

**Published:** 2008-05-13

**Authors:** Herbert L Needleman

## Abstract

Why did the EPA dismiss a highly respected neurotoxicologist as chair of its external review panel on the fire retardant deca? Pioneering lead researcher Herbert Needleman, MD, argues that the answer has little to do with science.

For researchers who operate at the intersection of basic biology and toxicology, following the data where they take you—as any good scientist would—carries the risk that you will be publicly attacked as a crank, charged with scientific misconduct, or removed from a government scientific review panel. Such a fate may seem unthinkable to those involved in primary research, but it has increasingly become the norm for toxicologists and environmental investigators. If you find evidence that a compound worth billions of dollars to its manufacturer poses a public health risk, you will almost certainly find yourself in the middle of a contentious battle that has little to do with scientific truth (see Box 1).

**Box 1. A Battle-Tested Veteran in the Fight for Scientific Integrity**

**Liza Gross**

Herbert Needleman is no stranger to the smear tactics of industry. Needleman, a professor of psychiatry and pediatrics at the University of Pittsburgh, began to document the health effects of low lead exposure in the early 1970s. His groundbreaking work—which industry fought tooth and nail—clearly demonstrated lead's toxic effects on children, providing critical evidence for regulations to eliminate lead from gasoline and interior paints, and to lower the blood lead standard for children.

Concerned that blood lead levels in an older child would not reflect early exposures, Needleman developed a method to evaluate discarded baby teeth (both teeth and bone accumulate lead) for a more accurate history of past lead exposure. He found that inner-city children had higher lead levels than children living in the suburbs, even though none of the children showed signs of lead poisoning [5]. When Needleman presented his findings at a 1972 meeting of lead researchers, he was surprised by the venomous nature of attacks by industry scientists leveled at any researcher who dared present evidence that lead could cause harm at low doses.

Needleman continued his work and found that children with elevated tooth lead levels scored lower on a suite of cognitive tests measuring IQ, speech, and language skills. He published his results in a 1979 landmark study showing that early childhood exposure to low levels of lead could compromise a child's intellectual performance and behavior, again, without evidence of lead poisoning [6,7]. Six months later, Needleman received a call from a representative at the International Lead Zinc Research Organization, a nonprofit trade organization that conducts research on behalf of the lead and zinc industry, asking for his data. He declined.

The attacks began soon after, starting with a *Pediatrics* paper criticizing Needleman's 1979 study [8], followed by charges that the work was flawed in testimony before the EPA [9]. After reviewing the charges and original work, the EPA confirmed Needleman's findings [10]. Then, in 1991, two psychologists who provided expert testimony on behalf of the tetraethyl lead industry accused Needleman of scientific misconduct. One of the psychologists, Claire Ernhart, had written the critical *Pediatrics* paper and testified against his study before the EPA. The attorney who filed the complaint with the NIH Office of Research Integrity worked for a firm with links to the Ethyl Corporation of America, the major manufacturer of tetraethyl lead.

The University of Pittsburgh Medical School began a preliminary investigation of the charges, but denied Needleman's request for open hearings. Needleman sought the support of the faculty assembly, which unanimously voted for open hearings, filed a complaint in federal court, and had the support of 400 independent scientists calling on the chancellor to open the hearings. The university acceded. After a 2-day hearing, and months of deliberation, the committee released a unanimous decision: there was no evidence of scientific misconduct [11]. Thanks to Needleman's pioneering efforts to reduce the hazards of lead [7], average blood lead levels of children in the United States dropped an estimated 78% from 1976 to 1991 (http://www.hhs.gov/asl/testify/t960501b.html). Whether other defenders of public health will be spared a similar path may ultimately depend on stronger laws to safeguard scientific integrity—and public health—from the undue influence of industry.

The latest example of this trend involves decabromobiphenyl ether, a polybrominated diphenyl ether (PBDE) commonly known as deca. A widely used fire retardant, deca has been brought under close scrutiny by a growing number of reports of its toxicity. Increased levels of the agent have been found in the bodies of young children. To respond to this, the Environmental Protection Agency (EPA) convened a panel of experts in 2006 to determine the state of risk that accompanied its use and appointed Deborah Rice to chair the panel of outside experts. Rice, who had held an appointment as a senior toxicologist at the EPA, and who is now a toxicologist for the state of Maine, has won wide respect for her studies of neurotoxins. Indeed, in 2004, the EPA recognized her with one of its most prestigious scientific awards for “exceptionally high-quality research into lead's toxicity” (for information on the award, see http://es.epa.gov/ncer/staa/annual/2004/staa_faq.html).

Under her chairmanship, the PBDE report was completed and submitted in February of 2007. The American Chemistry Council (ACC), a chemical industry trade group, did not elect to contest the statements of the report; it chose instead to accuse Rice of bias against the use of deca and to pressure the EPA to dismiss her from the panel. In a letter to EPA Assistant Administrator for Research and Development George Gray on May 3, 2007, the ACC argued that Rice's appointment represented a conflict of interest and “might lack the impartiality and objectivity necessary to conduct a fair and impartial review of the data,” based in part on testimony she gave to the Maine State Legislature describing the dangers of deca-BDE and advocating a state mandate to phase out its use (for more information on her dismissal, see: http://energycommerce.house.gov/Press_110/110-ltr.031308.EPA.BPA.pdf) [1].

The EPA, without examining or contesting the charge of bias, complied. Rice was fired. The next formal act of the EPA was to remove all of her comments from the written report and completely erase her name from the text of the review. There is now no evidence that she ever participated in the EPA proceedings, or was even in the room. The only indication that another reviewer had served on the panel was this note in the “revised” report: “Notice: EPA modified this report in August 2007 to include only four of the five reviewers' comments. One reviewer's comments were excluded from the report and were not considered by EPA due to the perception of a potential conflict of interest.”

In the interests of full disclosure, I should say that I have been a friend and admirer of Deborah Rice for many years. Our friendship extends back at least to the late 1970s, when we both were studying the toxicity of lead at low levels, she in primates at the EPA and I in children at Harvard Medical School. She is particularly memorable to me because she knew her stuff and brooked no vagueness or dissembling. She moved the leading edge of neurotoxicology forward by replacing rodents with primates to study the behavioral effects of lead [2]. Her colony of monkeys was carefully maintained until they reached the age of 26 years, enabling studies of the effects of lead on aging. This alone was a heroic effort. Her broad sphere of interest combined with an acute and critical mind has gained wide respect in the toxicology community. She was a natural choice as chair of the panel examining PBDE.

It appears that some in Congress agree. In a March 13 letter to EPA Administrator Stephen Johnson, Rep. John Dingell, who is overseeing a congressional investigation into conflicts of interest in EPA scientific review panels, asked why the agency would remove Rice as chair of an external review panel at the request of the chemical industry (http://energycommerce.house.gov/Press_110/110-ltr.031308.EPA.BPA.pdf). “The ACC does not assert that Rice has any pecuniary interest in the human health assessment at issue,” Dingell writes, “and therefore seems to argue that scientific expertise with regard to a particular chemical and its human health effects is a basis for disqualification from a peer review board. This does not seem sensible on its face.” He goes on to argue that the EPA's routine reliance on chemical industry employees and representatives for scientific review, “together with the dismissal of Rice, raises serious questions with regard to EPA's conflict of interest rules and their application.”

Rice's experience, like that of so many other researchers who find themselves locked in battle with industry giants, reveals the inherent disconnect between the interests of science and those of commerce. The scientific community is governed by its own rules, codified in the 1940s by Robert Merton, the distinguished Columbia University sociologist, as four normative standards for scientific conduct [3]. Science is *universal*: the rules apply every where to every one. Science is *communal*: the fruits of science belong to everyone. Science is *disinterested*: the discoveries of science are not affected by personal gain, ideology, or any cause but the truth. Finally, science is regulated by *organized skepticism*: scientists do not accept the claims of a hypothesis unless both its methods and evidence have been rigorously vetted. The conclusions drawn by scientists rely on these normative standards. However, these criteria do not stand up well in the face of the ethos of commerce. There is an unavoidable tension between the interests of commercial entities fueled by corporate profit reports and those true scientists whose motivation is curiosity, peer recognition, and societal benefit.

Merton's standards seem to have had some force until 1980, when the Bayh-Dole Act was passed. This act permitted scientists and universities to patent their discoveries, and it opened the floodgates to financial interests. The ethos of university science shifted. It allowed scientists to get rich by patenting their discoveries and partnering with the private sector while being funded by government grants. More and more university scientists receive varying degrees of industry support and increasingly participate in regulatory activity. I realized this when I was appointed to an EPA advisory panel on human testing with pesticides. The chairman was a former EPA scientist who left the agency to start a commercial firm to test pesticide toxicity. The majority of members on that panel had some connection to industry. It was easy to identify most of those members who had industrial sponsorship by the tendentious quality of their arguments.

This disparity in values is displayed in clear relief in the Deborah Rice case. This is not to say that scientists are oblivious to the attraction of money. But it used to be that most people who opted for a career in the laboratory understood that they were accepting a modest lifestyle. Industry scientists have a different value system. Faced with a critical report by the deca panel, the first response of the bromine industry was to protect their bottom line and get Rice fired. The EPA, whose reputation for independence is not enviable, saluted and not only ejected Rice, but also eliminated any trace of her contribution. Does this strike a familiar note? Some notorious undemocratic regimes have cleansed history and science when the truth did not serve their purposes. One is reminded of the rewriting of history during the Stalin regime.

The newspapers regularly detail examples of EPA cupidity. As I write this, today's *New York Times* (March 13, 2008) carries this story: “The Environmental Protection Agency announced a modest tightening of the smog standard… overruling the unanimous advice of its science advisory council for a more protective standard.” [4]. And so it goes.

I recite this sordid affair because it displays the ethical insouciance of industry, and of those PhDs who wear the caps and gowns of the academy, while they embrace the mores of the marketplace.

Deborah Rice is widely admired by her colleagues for her intelligence, integrity, and moral compass. She will withstand this insult and continue to contribute to the public welfare. Science and public health badly need more people like her.

## References

[pbio-0060129-b001] Kneiss S (May 3, 2007).

[pbio-0060129-b002] Rice DC (1992). Behavioral effects of lead in monkeys tested during infancy and adulthood. Neurotoxicol Teratol.

[pbio-0060129-b003] Merton RK, Storer NW (1942). The normative structure of science. The Sociology of Science.

[pbio-0060129-b004] Wald ML (March 13, 2008). Environmental agency tightens smog standard. The New York Times.

[pbio-0060129-b005] Needleman HL, Tuncay O, Shapiro IM (1972). Lead levels in deciduous teeth of urban and suburban American children. Nature.

[pbio-0060129-b006] Needleman HL, Gunnoe C, Leviton A (1979). Deficits in psychological and classroom performance of children with elevated dentine lead levels. N Engl J Med.

[pbio-0060129-b007] Needleman HL, Schell A, Bellinger D, Leviton A, Allred EN (1990). The long-term effects of exposure to low doses of lead in childhood. An 11-year follow-up report. N Engl J Med.

[pbio-0060129-b008] Ernhart CB, Landa B, Schell NB (1981). Subclinical levels of lead and developmental deficit: a multivariate follow-up reassessment. Pediatrics.

[pbio-0060129-b009] Ernhart CB (April 15, 1982). Testimony before the Environmental Protection Agency.

[pbio-0060129-b010] Environmental Protection Agency (March 1986). Review of the National Ambient Air Quality Standards for Lead: Assessment of Scientific and Technical Information.

[pbio-0060129-b011] Putka G (May 27, 1992). Professor's data on lead levels cleared by panel. Wall Street Journal.

